# Exotic pets in Ireland: 1. Prevalence of ownership and access to veterinary services

**DOI:** 10.1186/s13620-021-00190-6

**Published:** 2021-05-26

**Authors:** Matt Goins, Alison J. Hanlon

**Affiliations:** School of Veterinary Medicine, University College Dublin, Dublin, Ireland

**Keywords:** Exotic pet, Responsible pet ownership, Animal welfare, Veterinary services, Veterinary profession

## Abstract

**Background:**

There has been increasing concern expressed about the welfare of exotic pets worldwide. For the purposes of this article, an exotic pet is considered to be a non-domesticated species, where there are knowledge gaps on good practice (minimum standards of care), veterinary diagnostics and treatments. The categories of exotic pets included in this study were: small mammals (< 20 kg), large mammals (> 20 kg), birds, reptiles, amphibians, fish and invertebrates. Dogs, cats, rabbits, hamsters, gerbils, guinea pigs, mice, rats, and ferrets were excluded from the study. An online survey of pet owning staff at University College Dublin was conducted between July and August 2020 to provide the first empirical data for Ireland. In this pilot study (the first in this thematic series) we aim to characterise exotic pet ownership, the challenges and benefits of keeping exotic pets, and access to veterinary services from the pet owner’s perspective.

**Results:**

Using an online survey this pilot study gathered evidence from 83 domestic and 32 exotic pet owners within the staff (*n* = ~ 3600 FTE) at University College Dublin, the largest third level institution in Ireland. The prevalence of exotic pet ownership was determined to be 34.4 % of households with more than 10 % of pet owning households owning only exotic pets. Fish (*n* = 15), reptiles (*n* = 13) and birds (*n* = 8) were the most common types of exotic pets. Thirty-four per cent of exotic pet owners never sought veterinary services, the most common reasons were due to lack of local veterinary specialists (*n* = 10) and good owner knowledge (*n* = 8). However identifying appropriate guidance on the animals’ needs was a common challenge for exotic pet owners (*n* = 13). A reasonable monthly cost of caring for an exotic ranged from €20–180, depending on the species.

**Conclusions:**

This pilot study contains important implications for veterinary education to support the veterinary community with providing services to the exotic pet owning community. Policy issues with exotic pet ownership also need to be considered and further research into the proposed strategies to support the health and welfare of exotic pets should be carried out such as the introduction of white lists and guidelines on responsible pet ownership.

**Supplementary Information:**

The online version contains supplementary material available at 10.1186/s13620-021-00190-6.

## Background

There is no generally accepted definition of an exotic pet across national or international veterinary bodies [1–3]. For example, the British Zoological Veterinary Society states that exotic pets “*are strictly defined as being one of the non-domestic animals, i.e. not cat, dog, cow, horse, sheep, pig, or commercial chicken*” while noting that many would not consider pets such as rabbits or guinea pig as exotic despite including them in their definition [1] and the American Veterinary Medical Association defines exotic pets as “*a wide range of pet species other than domestic dogs, cats, and equids, which may be native or non-native to the United States*” [2]. Differences in nomenclature are evident between Europe and North America as well. For example, ‘*pocket pets*’ are used to refer to rodents, ferrets, and rabbits as well as animals such as hedgehogs and sugar gliders [4]. For the purposes of this pilot study, an exotic pet was defined as a non-domesticated species, where there are knowledge gaps on good practice (minimum standards of care), veterinary diagnostics and treatments. Seven categories of exotic pet were included: small mammals (< 20kg), large mammals (> 20kg), birds, reptiles, amphibians, fish and invertebrates. Dogs, cats, rabbits, hamsters, gerbils, guinea pigs, mice, rats, and ferrets were excluded from the study.

The prevalence of exotic pet ownership is increasing worldwide such as in the United Kingdom [5], Canada [6], Australia [7, 8], and the United States [9], as well as across Europe [9, 10], Asia [9], and South America [9]. Ownership of exotic pets is reported to be 7.1 % [11], 24.1 % [12], and 36 % [13] of households in the UK, US, and Canada respectively, however these studies use a less restrictive definition of exotic pet than this pilot study. The prevalence of exotic pet ownership in the Republic of Ireland is undocumented. Pet choices may be influenced by portrayal of animals in the media and in the case of exotic pets, the popularity of broadcast series such as Netflix’s Tiger King as well as Teenage Mutant Ninja Turtles (turtles), Finding Nemo (clownfish and blue tangs), and the Harry Potter film series (owls) [14] may reflect the zeitgeist.

There are concerns related to the welfare of exotic pets in the areas of “behavioural or interactive restriction” and “anxiety, pain, fear, or distress” [15]. For example those that relate to environment-focused activities of exploration and food acquisition, play behaviour or interactive behaviour with conspecifics [16] and those associated mainly with sensory inputs including “anxiety, fear, panic, frustration, anger, helplessness, loneliness, boredom and depression” [16]. However few studies [17] have been conducted to investigate the owners’ perspective of keeping exotic pets, within the context of animal welfare, and barriers to accessing veterinary services.

The aims of this pilot study were to calculate a prevalence of exotic pet ownership within the respondent pet owning population in University College Dublin, Ireland, to characterise the availability and ease of procuring veterinary services for their exotic pets, and to gather information relating to policy proposals that could enhance the health and welfare and strengthen the human-animal bond of exotic pets.

## Results

### Respondent demographics

A total of 104 individuals accessed the survey. One respondent did not consent to the survey, a further eight of those that consented did not answer any questions, and two respondents did not own any pets, resulting in 93 (89.4 %) individuals completing at least the first section. This is from a pool of approximately 3600 FTE employees, which in a previous study showed a prevalence of dog ownership of 47 % [18].

Respondents comprised 61 (65.6 %) domestic only pet owners, 10 (10.8 %) exotics only pet owners, and 22 (23.7 %) pet owners who owned both domestic and exotic pets (Table 1). On average, it took five minutes to complete the survey.

**Table 1:** Percentage prevalence, number and type of domestic and exotic pets owned by survey respondents in University College Dublin, Ireland

Category	Prevalence % (n)
**Domestics (overall)**	89.24 (83)
Dogs	56.99 (53)
Cats	51.61 (48)
Rabbits	5.37 (5)
Hamsters	0.00
Guinea Pigs	5.37 (5)
Gerbils	0.00
Mice/Rats	2.15 (2)
Ferrets	0.00
**Exotics (overall)**	34.41 (32)
Small Exotic Mammals (SEM)	5.37 (5)
Large Exotic Mammals (LEM)	1.08 (1)
Birds	8.60 (8)
Reptiles	13.98 (13)
Amphibians	0.00
Fish	16.13 (15)
Invertebrates	5.37 (5)

### Differences in pet ownership by highest household educational attainment

**Fig. 1:**
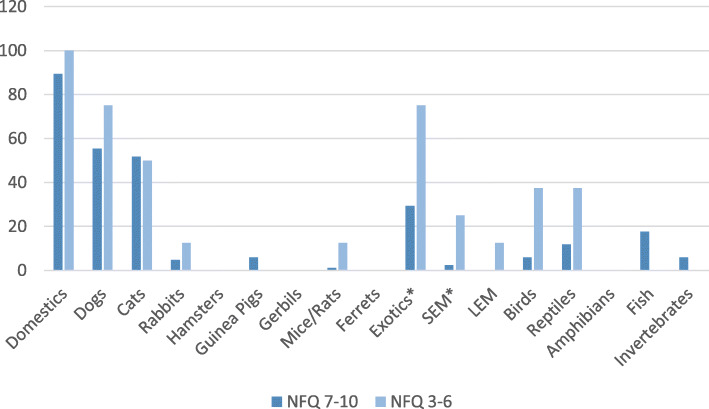
Prevalence of domestic and exotic pet ownership by highest level of educational attainment per household, NFQ 7–10 (*n* = 85) and NFQ 3–6 (*n* = 8). Significant results are denoted by an asterisk

The highest level of education (on the 10-level Irish National Framework of Qualification scale) in respondents’ households ranged from NFQ level 3 (Junior Certificate) to NFQ Level 10 (Doctoral Degree). Differences in pet ownership were compared between households where the highest level of educational attainment corresponded to NFQ level 7 or higher (*n* = 85) and those below NFQ level 7 (*n* = 8). Significant differences were found in the prevalence of exotic pet ownership overall (*p* = 0.015) as well as the prevalence of ownership of small exotic mammals specifically (*p* = 0.036) with households whose highest level of educational attainment was below NFQ level 7 being more likely to own these types of pets (Fig. 1).

### Differences in pet ownership by respondent housing type

**Fig. 2:**
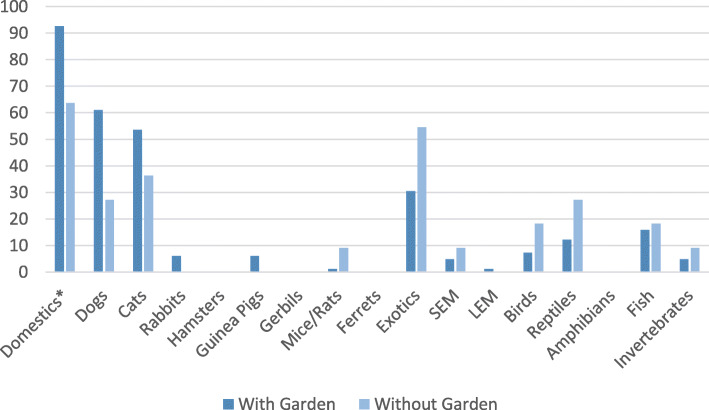
Prevalence of domestic and exotic pet ownership by living situation, divided by presence or absence of a garden. Significant results denoted by an asterisk

Respondents largely lived in houses with a garden (*n* = 82), followed by apartments (*n* = 5), houses without a garden (*n* = 4), and shared apartments (*n* = 2). The latter three categories of dwelling were pooled due to the small sample sizes to compare respondents living in dwellings either with (*n* = 82) or without a garden (*n* = 11). Domestic pet ownership was significantly higher in those with a garden (92.68 %) than those without a garden (63.63 %) (*p* = 0.016) (Fig. 2).

### Differences in pet ownership by household presence or absence of children

**Fig. 3:**
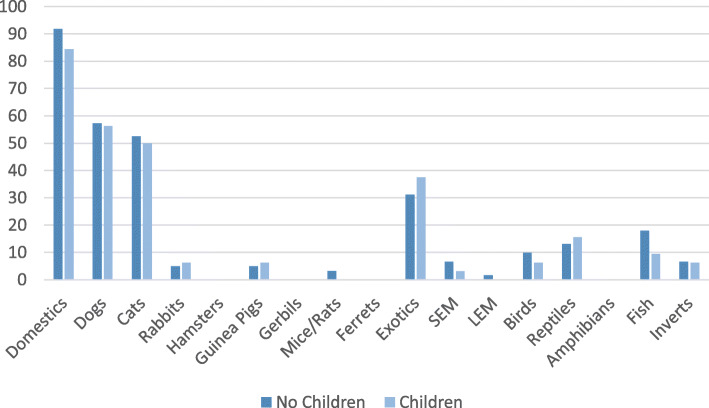
Prevalence of domestic and exotic pet ownership by presence or absence of children in the household

The age demographic of respondent households was divided into three categories (adults (aged > 18), young adults (aged 13–18), and children (aged up to 12)). Adult only households (*n* = 61) and households with children (*n* = 32) were then compared but there were no significant differences between these cohorts and categories of pet (Fig. 3).

**Fig. 4:**
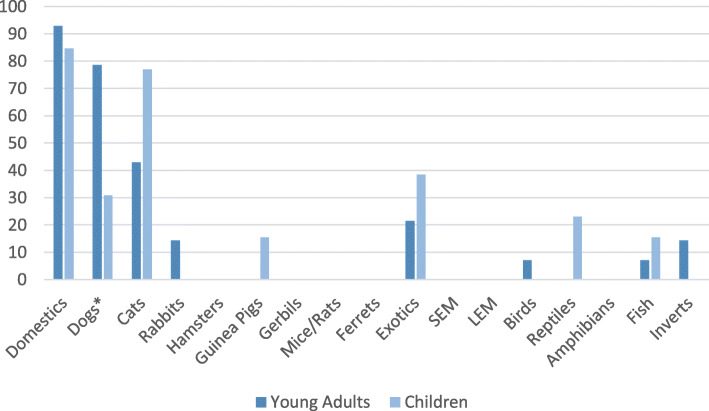
Prevalence of domestic and exotic pet ownership by presence of young adults (aged 13–18, *n* = 14) or children (aged up to 12, *n* = 13) within a household. Significant differences denoted by an asterisk

Within households containing children, households with only young adults (aged 13–18, *n* = 14) were compared to households with only children (aged up to 12, *n* = 13). Households with only young adults were found to be significantly more likely to own dogs (*p* = 0.02) (Fig. 4).

### Access to veterinary services

Of the 32 respondents that owned exotic pets, 22 (68.75 %) relayed information about their experiences in 2019 of seeking veterinary services. Eleven respondents never sought veterinary services for their exotic pet(s). Of the remaining 11 respondents, four had sought veterinary services for a bird, four for a reptile, two for a small exotic mammal, and one for a fish.

Additionally, 11 (50 %) of the exotic pet owners indicated that they were first time owners of this type of exotic pet. No statistically significant difference was found between experience level (first time owners vs. more experienced owners) and likelihood to access veterinary services during 2019. For both groups, ‘routine check-up’ was the most common reason respondents had sought access to veterinary care.

Respondents were also asked about factors that would reduce the likelihood of accessing veterinary services. The most common responses were a lack of veterinary clinics nearby that specialised in their exotic species (*n* = 10, 45.45 %) and that the respondent possessed good knowledge of the species precluding the need to access veterinary services (*n* = 8, 36.36 %).

### Benefits and challenges of exotic pet ownership

Respondents were asked to outline the main perceived benefits and challenges of exotic pet ownership. The most commonly cited benefits included that the pet is friendly, affectionate, or easy-going (*n* = 8), that the pet was calming to the owner (*n* = 7), and that exotic pet ownership offered a different view on animals (*n* = 6). Additionally, several respondents noted the increased longevity of certain types of exotic pet when compared to common domestic species as well as the idea that it was impossible for their exotic pet to experience a negative interaction with another pet in public. The most commonly cited challenge of exotic pet ownership was identifying and providing for the specific needs of each species (*n* = 13), finding a veterinary practitioner with experience in exotic pet medicine (*n* = 5), and lack of space to allow natural behaviours (*n* = 5) were also notable responses.

Respondents were also asked what a reasonable cost was per month to look after their type of exotic pet, benchmarked against the reported cost of care for a dog or cat given as approximately €80 per month [19]. Responses ranged from €20–180, with mean results of €100 per month for small exotic mammals such as an African pygmy hedgehog, €32 for birds such as parakeets and cockatiels, €43 for reptiles such as a panther chameleon and a bearded dragon, and €24 for fish.

### Development of strategies to support improved health and welfare of exotic pets

The majority of respondents (*n* = 20, 90.91 %) agreed that there should be national guidelines developed to support the improved health and welfare of exotic pets. Suggested strategies included guidelines outlining the basic needs and healthcare of exotic species, licensing and testing of owner competency (e.g. knowledge of husbandry), and a register of veterinary practitioners specialising in exotic species in order to simplify searching for veterinary services.

## Discussion

A key challenge of the survey was gaining access to exotic pet owners in Ireland. University College Dublin is the largest third level institution in the country with approximately 3600 FTE staff, providing a wide ranging demographic. An invitation to participate in the online survey was contained in two staff e-zines, circulated during the summer. The impact of the Covid-19 pandemic on working life is likely to have reduced engagement with the survey. Overall prevalence of pet ownership in the Republic of Ireland is unknown, though there is data supporting the prevalence of dog ownership to be 47 % at University College Dublin [18]. However, overall prevalence of pet ownership in other western countries is around 41–65 % [8, 11-13].

### Prevalence of exotic pet ownership

This pilot study demonstrates that exotic pet ownership amongst survey respondents is relatively common, considering 34.4 % of pet owning households reported ownership of at least one exotic pet and more than 10 % of households reported owning only exotic pets. This view is further supported by many of the exotic pet categories in this pilot study possessing higher levels of prevalence of ownership than every domestic category that was not a cat or dog. This takes on increased importance in light of the ongoing COVID-19 pandemic, which is generally accepted as having originated from a wildlife reservoir [20]. From a One Health perspective considering that 60.3 % of emerging infectious diseases worldwide are zoonotic [21], with pets being a risk factor for illness caused by various pathogens [13], and the pet trade producing several high profile invasive species [9] has resulted in the FVE calling for countries to institute positive lists or ‘white lists’ of animals to be allowed to be kept as companion animals/pets, such as those instituted by Belgium and the Netherlands [3].

Several demographic differences characterised pet ownership (domestic and exotic pets) including household educational attainment level, presence of a garden, and presence/absence of children in the household. For example survey respondents owning an exotic pet tended to have a lower educational attainment than domestic pet owners, ownership of domestic pets was greater in households with a garden, and ownership of dogs corresponded to households with no children. The small sample of exotic pet owners in our survey negates drawing clear conclusions about the societal demographic most likely to keep non-traditional pets.

### Access to veterinary services

It is currently considered best practice for dogs and cats to access veterinary services at least once per year for an annual health check [22, 23] and according to the PDSA [19], 92 % of dogs and 84 % of cats in the UK are currently registered with a veterinary clinic. Comparable figures for the Republic of Ireland are unavailable. In contrast only 50 % of exotic pet owners in this survey reported seeking a veterinary consultation in 2019. Whilst routine check-ups were the most common reason for owners seeking veterinary advice, barriers to accessing veterinary services included the lifespan of the animal and a perception of a lack of species-specific competency in veterinary services. Furthermore owners indicated a good knowledge of their exotic species, negating the need to visit a veterinary professional for advice. This raises potential welfare implications for exotic pets that are self-treated by their owners, as has been reported for traditional companion animals (e.g. NSAID administration in cats) [24, 25, 26, 27]. A lack of guidelines on husbandry was also cited as a challenge of exotic pet ownership.

### Alleviating the challenges of exotic pet ownership

The challenges previously noted by respondents are well supported by the literature, which has found that lack of information specific to species was a particular animal welfare concern [15] as well as the difficulty in locating appropriate veterinary services [28, 29]. Additionally, respondents indicated cost-sensitivity in terms of what is “reasonable” to spend on an exotic pet in comparison to a domestic pet. This is at odds with the expectation of having access to specialist veterinarians and the additional professional costs of gaining a specialist qualification. For example, the American Board of Veterinary Practitioners requires four or more years specialty experience via internships, residencies, and other approved training programmes [30] with the applicant generally expected to be paid at a rate lower than a general practitioner and the postgraduate certificate in exotic animal practice available in the United Kingdom by Harper Adams University is expected to take two years to complete at a cost of £9,495 [31].

A good counterbalance to these challenges are a number of the proposed strategies by respondents. Guidelines of care, training and licensing of owners, and a register of qualified veterinary practitioners would help to address these concerns.

## Conclusions

One third of respondents owned an exotic pet. This has implications for veterinary education to support the veterinary community with providing services to the exotic pet owning community. The development of guidelines on responsible ownership of exotic pets and codes of practice on animal care are required to address owner concerns.

Policy issues with exotic pet ownership need to be considered. Examples to be explored include development of a list of non-domesticated species that are permitted to be kept as pets such as a positive or white list as instituted by the governments of Belgium [3], the Netherlands [3], and Luxembourg [32] or implementation of a licensing systems for exotic pet owners.

Additionally, research into the proposed strategies to support the health and welfare of exotic pets are required to examine their impact on pet owners, the veterinary profession, and the welfare of the animals involved.

## Methods

### Survey design

An online survey was designed to characterise exotic pet ownership including the owners’ perspective to the benefits and challenges of caring for exotic pets and to examine veterinary services by exotic pet owners in University College Dublin, Ireland. The survey consisted of 16 questions, divided into three sections: information about the respondent’s household, exotic pet ownership, and exotic pet health.

Section one consisted of consent to participate in the pilot study and questions about the respondent’s household such as the number of people living in their household, age profiles, type of dwelling (and presence or absence of a garden), highest educational level attained by a household member, the number and types of domestic companion animals owned, and whether there were any exotic pets kept in the household. This final question was used to exclude respondents that did not own exotic pets from answering sections two and three.

Section two consisted of a single question asking the respondent to enumerate the exotic pets contained within their household categorised as: small exotic mammals (< 20 kg), large exotic mammals (> 20 kg), birds, reptiles, amphibians, fish, and invertebrates.

Section three consisted of 11 questions asking the respondent about their most recent access to veterinary services, how many times veterinary services were accessed in 2019, the perceived benefits and challenges of owning that type of exotic pet, and owner attitudes and suggestions with regard to various policy proposals around exotic pet ownership.

The survey was published online using SurveyMonkey® and was open for responses from 15 July  to 5 August 2020. An invitation to complete the survey was distributed by University College Dublin in their staff e-zine on 15 July and 29 July 2020. The study population of staff at University College Dublin comprised approximately 3600 FTE Employees, a diversity of roles (academic, technical, support and administration) and levels of educational attainment. A copy of the survey can be found in the Supplementary Materials.

### Data handling and analysis

Data were exported from SurveyMonkey® into Microsoft Excel (2013). R version 3.6.1 and R Studio version 1.2.1335 were used for data cleaning and transformation, data visualisation, generating descriptive statistics, and for all statistical analyses. Microsoft Excel (2013) was also used to generate graphs. To test for statistically significant differences in the frequencies of responses between independent cohorts, a Pearson’s Chi-Squared test or Fisher’s exact test was conducted depending on sample size. Comparisons were made between the responses of respondents living in houses with gardens and those without gardens, respondents whose household highest educational attainment fell between NFQ Level 3 and 6 and those whose household highest educational attainment fell between NFQ Level 7 and 10, and households that included children and those whose households contained only adults. The significance threshold for statistical analyses was *p* < 0.05.

## Supplementary Information

**Additional file 1.** Exotic pet owners survey

## Data Availability

The data generated during this study are available from the corresponding author on reasonable request.

## Abbreviations

FTE: Full Time Equivalent
NFQ: National Framework Qualification
