# Supplementary material for: Exotic pets in Ireland: 1. Prevalence of ownership and access to veterinary services
Source: Ir Vet J. 2021 May 26;74:14. doi: 10.1186/s13620-021-00190-6 (PMC8153849; doi:10.1186/s13620-021-00190-6)
Supplement: Supplementary file 1 — Additional file 1. Exotic pet owners survey [file 13620_2021_190_MOESM1_ESM.pdf]

Exotic Pet Owners Survey

Welcome to the Exotic Pet Owner Survey!

Dear Survey Participant,
I am a veterinary student at UCD, conducting a Summer Research Award project with Associate Professor Alison Hanlon. The project focuses on exotic pet ownership in Ireland, common issues with exotic pets and access to veterinary care. Exotic pets are defined for the purposes of this study as a household animal/pet that is not a dog, cat, rabbit, hamster, gerbil, guinea pig, mouse, rat, or ferret.

The survey has three sections: Information about your household (5 questions; all participants), exotic pet ownership (1 question; exotic pet owners only), and exotic pet health (11 questions; exotic pet owners only). No identifiable data is requested and so responses are anonymous. The survey should take less than 10 minutes to complete. We would really appreciate your help us gain valuable insight into exotic pet ownership in Ireland.

The survey closes on 5 August 2020.

Sincerely,
Matt Goins 15200869@ucdconnect.ie

1. I give my consent to participate in this project and understand that the data will be used for research purposes and to support the development of veterinary education.

◯ Agree

◯ Disagree

Exotic Pet Owners Survey

About you and your household

* 2. Where do you live?

- House with garden
- House without garden
- House-share (with non-family members)
- Other (please specify)
- Apartment
- Apartment-share (with non-family members)

* 3. How many people are in your household?

Adults (more than 18 years old)

Young adults (13-18 years old)

Children (up to 12 years old)

4. What is the highest educational level attained (or equivalent) by someone in your household?

- NFQ Level 3 (Jr. Cert)
- NFQ Level 4/5 (Leaving Cert)
- NFQ Level 6 (Higher/Advanced Certificate)
- NFQ Level 7 (Ordinary Bachelor’s)
- Other (please specify)
- NFQ Level 8 (Honour’s Bachelor’s)
- NFQ Level 9 (Master’s)
- NFQ Level 10 (Doctoral)

* 5. How many of the following type of companion animals are in your household?

Dogs

Cats

Rabbits

Hamsters

Guinea pigs

Gerbils

Mice/rats

Ferrets

* 6. Are there any exotic pets in your household (e.g. reptiles, amphibians, birds, fish etc)?

- Yes
- No

About the exotic pets in your household

* 7. How many of the following categories of exotic pets do you have in your household?

Small exotic mammals (<20kg) e.g. chinchilla	
Large exotic mammals (>20kg) e.g. pot bellied pig	
Birds	
Reptiles	
Amphibians	
Fish	
Invertebrates (e.g. insects)	
Other (please specify)	

Your <u>Most Recent</u> Access to Veterinary Services

To answer the following questions, please focus on the exotic pet species that you (or a member of your household) have most recently taken to a vet clinic

* 8. What type of exotic pet have you most recently taken to a vet clinic?

* 9. Is this the first time that either you or a member of your household have owned this type of exotic pet?

- Yes
- No
- Other (please specify)

* 10. Approximately how many years have you owned this exotic pet?

1 year (or less) 15 years 30 years

* 11. Based on your experience in 2019, how many times did you (or someone in your household) require access to veterinary services for this exotic pet?

* 12. What was the main reason for your last vet consultation for your exotic pet?

- Routine check-up
- Behavioural issues
- Nutritional issues
- Other health-related conditions (please specify)

* 13. Which factors reduce the likelihood of taking your exotic pet to the vet? (Tick all that apply)

- [ ] Vet care is too expensive
- [ ] There are no nearby vets that specialise in exotic pets
- [ ] The natural lifespan of the exotic pet is too short to seek veterinary care
- [ ] I have good knowledge of caring for exotic pets and have less need to access vet services
- [ ] Other (please specify)

* 14. Apart from your local vet clinic, what other sources of information do you use for guidance in looking after your exotic pet? Tick all that apply.

- [ ] Internet
- [ ] Special interest group (e.g. Facebook)
- [ ] Biologist/Zoologist
- [ ] Other (please specify)

* 15. What are the 3 main challenges of owning this exotic pet species?

Issue 1

Issue 2

Issue 3

16. What are the 3 main benefits of owning this exotic pet species?

Benefit 1

Benefit 2

Benefit 3

* 17. In your opinion, what is a reasonable cost per month to look after this type of exotic pet?
For example, it is reported to cost approx. €80/month for a dog or cat (includes food, veterinary costs, kennelling during holiday periods etc).

€0 €100 €200

18. In your opinion should there be national guidelines or other strategy developed to support the improved health and welfare of exotic pets?

- ◯ Yes
- ◯ No
- ◯ If YES, what type of guidelines would be helpful?

Thank-you!

We’d like to thank you again for taking part in this project. If you are interested to find out more about UCD research on animal welfare, please go to: www.ucd.ie/animalwelfare
